# Immunomodulatory Effects of *Lycium barbarum* Polysaccharide Extract and Its Uptake Behaviors at the Cellular Level

**DOI:** 10.3390/molecules25061351

**Published:** 2020-03-16

**Authors:** Le Feng, Xiao Xiao, Jing Liu, Junyan Wang, Nan Zhang, Tao Bing, Xiangjun Liu, Ziping Zhang, Dihua Shangguan

**Affiliations:** 1 Key Lab of Ministry of Education for Protection and Utilization of Special Biological Resources in Western China, School of Life Sciences, Ningxia University, Yinchuan 750021, China; 2 Beijing National Laboratory for Molecular Sciences, Key Laboratory of Analytical Chemistry for Living Biosystems, CAS Research/Education Center for Excellence in Molecular Sciences, Institute of Chemistry, Chinese Academy of Sciences, Beijing 100190, China; 3 Institute of Chemistry, University of Chinese Academy of Sciences, Beijing 100049, China

**Keywords:** *L. barbarium* polysaccharide, immunomodulatory effects, polysaccharide uptake, extraction, physicochemical property

## Abstract

*Lycium barbarum* L. is a widely used functional food and medicinal herb in Asian countries. *L. barbarium* polysaccharides (LBP) are considered as one of the major medicinal components of *L. barbarium* fruit and exhibits a wide range of biological activities. Here, we investigated the immunomodulatory effects of LBP and its uptake behaviors at the cellular level. LBP was prepared by water extraction and ethanol precipitation, and divided into two fractions based on the molecular weight distribution by ultrafiltration (LBP > 10 kDa and LBP < 10 kDa). The physicochemical properties of LBP and LBP fractions were well characterized. The LBP > 10 kDa fraction greatly enhanced the viability of macrophages RAW264.7 cells and induced cell polarization, but had weak effects to other tested tumor cell lines and normal cell line. This fraction could regulate the production of NO, TNF-α, IL-6 and ROS in RAW264.7 cells, suggesting both pro-inflammatory and anti-inflammatory effects. The dye-labeled LBP could be internalized into all tested cell lines and accumulated in lysosomes. The internalization of LBP in RAW264.7 cells is mainly through the clathrin-mediated endocytosis pathway. The Caco-2 intestinal transport experiment demonstrated that the dye labeled LBP could be transported through the Caco-2 cell monolayer (mimic intestinal epithelium) through clathrin-mediated endocytosis. These results demonstrate the immunomodulatory effects of LBP and its effective uptake by macrophages and intestine.

## 1. Introduction

*Lycium barbarum* L. has been widely used as a functional food and medicinal herb in China and other Asian countries for centuries [1]. In recent years, thanks to its excellent nutritional value and pharmacological effects, it has received extensive attention and has been advertised as “super food” in Europe and North America [2]. *L. barbarium* polysaccharides (LBP) are one of the major medicinal components of *L. barbarium* fruit and exhibits a wide range of biological activities, such as antioxidant [3,4], neuroprotection [5,6], radioprotection [7], hepatoprotection [8,9], anti-osteoporosis [10], antifatigue [11], and immunomodulation [12,13,14,15,16]. It also has been reported that LBP are glycoprotein complexes or polysaccharide-protein complexes [1,12,17]. In recent years, with the rapid development of sugar chemistry and glycobiology, more and more Chinese medicine polysaccharides with outstanding biological activity have been reported one after another [18,19,20]. The biological activities of polysaccharides are mainly affected by their high-order structure, the linkage mode of main chain glycosidic bonds [21], molecular weight [22], degree of polymerization, degree of branching of side chains, monosaccharide composition and functional groups [23], etc. While the physicochemical properties and chemical structure of polysaccharides are also affected by the extraction and purification methods. Therefore, detailed extraction steps and structural characterization are necessary for reference comparison of the biological activity of the polysaccharide. 

As hydrophilic macromolecules, whether polysaccharides can be absorbed by oral administration is a controversial issue [24]. At present, oral administration is the only way to take LBP. However, there is still a lack of knowledge on whether and how LBP is absorbed by the gastrointestinal tract, and whether and how LBP enters the cells to exert biological effects. Because of the structural heterogeneity and the lack of chromophore, the quantitative study of the uptake behavior of polysaccharides by gastrointestinal tract and cells is very difficult. Fluorescence-based bioimaging technology has been widely used in the field of bioimaging because of its inherent high sensitivity, high selectivity, convenience and non-invasiveness [25], and has been used to track cellular uptake and endocytosis of polysaccharides [24,26]. Caco-2 cells are derived from human colonic adenocarcinoma cells and can undergo epithelial differentiation to form a single cell layer with similar structure and function to the intestinal epithelium [27]. It has been widely used in in vitro absorption experiments of oral drugs.

In this study, crude polysaccharide extract from *Lycium barbarum* L. was prepared by water extraction and alcohol precipitation, and the LBP was further separated by ultrafiltration to LBP > 10 kDa and LBP < 10 kDa fractions based on the molecular weight distribution. The monosaccharide compositions, molecular weights, fourier transform infrared spectroscopy (FTIR), chemical composition and elemental analysis of the samples were characterized. Based on this, the immunostimulatory properties and the uptake process of LBP were investigated. Furthermore, the absorption mechanism of LBP was also studied using a Caco-2 cell model.

## 2. Results and Discussion

### 2.1. Preparation and Characterization of LBP

**Figure 1:**
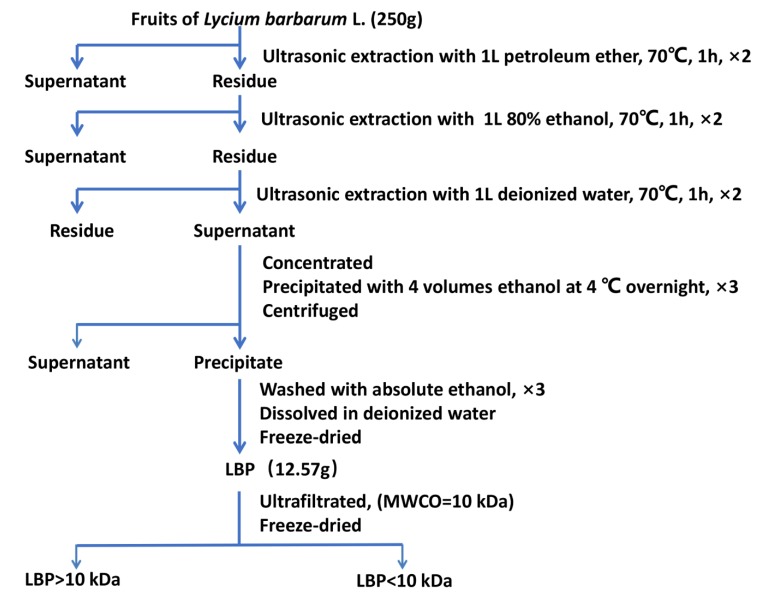
Extraction scheme and isolation process of LBP from *Lycium barbarum* L. fruit.

The crude polysaccharide (LBP) extraction process (Figure 1) was as follows: petroleum ether degreasing, 80% ethanol removing small molecular components, hot water extraction and ethanol precipitation. The yield of crude polysaccharide after freeze drying was 5.03%. Then, the LBP was fractionated using an ultrafiltration membrane (MWCO = 10 kDa) to produce a retention fraction (LBP > 10 kDa) and a dialysis fraction (LBP < 10 kDa) with yields of 57.18% and 26.29%, respectively.

Some functional groups of polysaccharides have characteristic infrared absorption. The FIIR spectrum of crude LBP is shown in Figure S1. Some peaks such as the strong and broad absorption peak near 3415.83 cm^−1^, the strong peaks at 1406.06 cm^−1^ and 1078.17cm^−1^ suggest the presence of polysaccharides. The strong absorption peak at 1612.44 cm^−1^ might belong to the absorption of C=O groups, which might be contributed by the reduction end portion of glycosides and the peptide bonds, because LBP is glycoprotein complexes.

The detailed data of LBP characterization were shown in Table 1. The molecular weight of natural resource polysaccharides has an important effect on its biological activity [28]. The molecular weight distribution of LBP, LBP < 10 kDa and LBP > 10 kDa was determined by high performance size exclusion chromatography coupled with multi-angel laser light scattering and refractive index detector (HPSEC-MALLS-RID), which is a technique for independent determination of the absolute molecular weights of polymers in solution by detecting the scattered light of polymer components [29]. The HPSEC chromatogram of LBP showed two overlapping peaks at elution times of 16.17 min and 19.02 min (Figure S2), with weight average molecular weight (Mw) of 51.88 kDa and 6.71 kDa, respectively. In order to investigate the effect of molecular weight on the biological activity of polysaccharides, we used an ultrafiltration membrane (MWCO = 10 kDa) to further divide LBP into two components based on its molecular weight distribution, which were designated as LBP > 10 kDa (Mw = 27.7 kDa) and LBP < 10 kDa (Mw = 6.99 kDa). The detail information of the number average molecular weight (Mn) and the polydispersity index (Mw/Mn) of three samples were summarized in Table 1. 

An analysis of monosaccharide composition is necessary for structural characterization and quality control of polysaccharides [30]. The monosaccharides composition was analyzed by 1-Phenyl-3-methyl-5-pyrazolone (PMP) pre-column derivation HPLC method, which can simultaneously determine neutral, acidic and basic monosaccharides released from polysaccharides [31]. As shown in Table 1, nine monosaccharides were detected from the three polysaccharide fractions, including fucose, rhamnose, arabinose, xylose, glucose, mannose, glucosamine, galacturonic acid and galactose. In addition, a small amount of ribose was detected in LBP and LBP < 10 kDa, not in LBP > 10 kDa. The monosaccharide compositions of LBP < 10 kDa and LBP >10 kDa were very different. Glucose was the dominant monosaccharide of LBP < 10 kDa, with a molar ratio of 47.27%. The LBP > 10 kDa was mainly consisted of arabinose, galactose and glucose in a molar ratio percentage of 27.76%, 13.37% and 11.68%, respectively. The high level of arabinose and galactose in LBP > 10 kDa is similar to previous report [1]. 

The protein contents of LBP < 10 kDa, LBP and LBP > 10 kDa were 0.47% ± 0.07%, 0.65% ± 0.14% and 1.10% ± 0.03%, respectively. The carbohydrate contents of LBP < 10 kDa, LBP and LBP > 10 kDa were 16.25% ± 0.24%, 26.58% ± 0.51% and 29.69% ± 0.80%, respectively. Elemental analysis of the LBP < 10 kDa, LBP and LBP > 10 kDa showed remarkable variations in the distribution of C, H, N and S. C element was the dominant element in the LBP extract. The levels of N and S remained similar in all samples. The C and H levels in LBP > 10 kDa were significantly higher than those in LBP < 10 kDa (*p* < 0.0001), which was consistent with the results of carbohydrate content.

### 2.2. Fluorescent Labeling of LBP

In order to understand the cellular uptake of LBP in macrophages and its transport mechanism in Caco-2 cell monolayers, LBP was covalently reacted with FITC or RBITC, respectively. The fluorescently labeled products were named LBP-F and LBP-RB, and their absorption peaks were confirmed by UV-visible spectroscopy (Figures S3 and S4) at 488 nm or 558 nm. The fluorescence substitution degree of LBP-F and LBP-RB were calculated to be 0.86% and 1.45%, respectively, based on the regression equation of FITC and RBITC (Figures S5 and S6). 

### 2.3. Immunomodulatory Activities of LBP In Vitro

**Figure 2:**
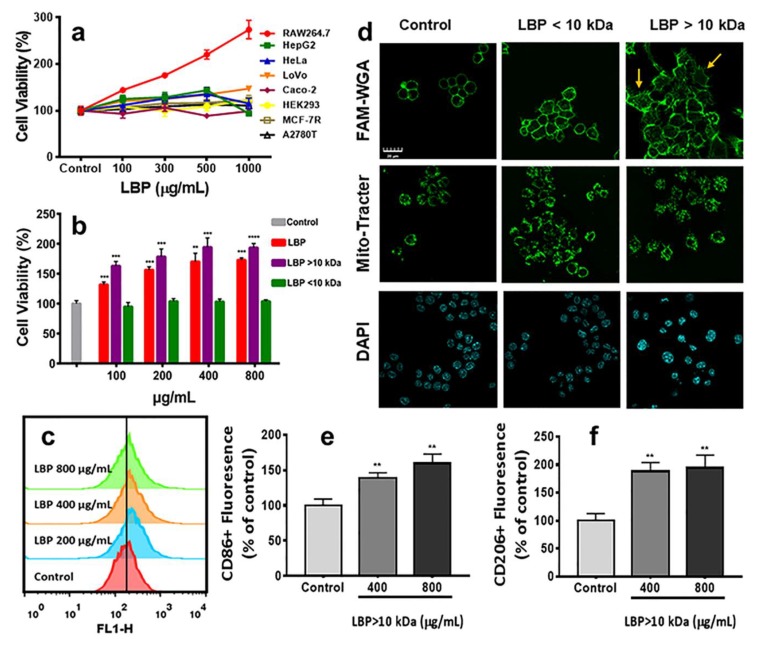
(**a**) Cell viability assay of different cell lines following the addition of LBP for 48 h; (**b**) cell viability assay of RAW264.7 cells treated with LBP, LBP < 10 kDa and LBP > 10 kDa for 48 h; (**c**) flow cytometry assay of CFSE distribution for investigating the effect of LBP on cell division (RAW264.7); (**d**) Effect of LBP < 10 kDa and LBP > 10 kDa on the morphology of macrophages, the yellow arrows indicate filopodia (scale bar, 20 μm); (**e**) Percentage of CD86 + RAW264.7 cells measured by flow cytometry after treatment by LBP > 10 kDa. (**f**) Percentage of CD206 + RAW264.7 cells measured by flow cytometry after treated by LBP > 10 kDa. Results were expressed as means ± SD, *n* = 3. * *p* < 0.05, ** *p* < 0.01, *** *p* < 0.001, **** *p* < 0.0001 vs. the control group.

In vitro effects of LBP were studied by CCK-8 assay in six tumor cell lines (HepG2, HeLa, LoVo, Caco-2, MCF-7R, A2780T), a human embryonic kidney cell line (HEK-293) and a macrophage-like, leukemia virus transformed murine macrophage cell-line (Raw264.7) that is commonly used as the model of mouse macrophages. As shown in Figure 2a, after treatment with LBP in the concentration range of 100~1000 μg/mL for 48 h, the viability of tumor cells was 94.3% ± 4.53%~147.51% ± 3.73%, indicating that LBP did not have anti-tumor activity on the tested cell lines. The viability of HEK-293 cells was 99.80% ± 12.09%~123.39% ± 5.34%, indicating that LBP had also no toxicity to normal cells. In contrast, with the increase in LBP concentration, the cell viability of macrophages increased significantly (*p* < 0.001), reaching the maximum cell viability of 274.07% ± 19.83% at 1000 μg/mL. Therefore, we further compared the effect of LBP, LBP < 10 kDa and LBP > 10kDa on RAW264.7 macrophages. As shown in Figure 2b, LBP and LBP > 10 kDa could significantly (*p* < 0.001) increase the cell viability of RAW264.7 cells in a concentration-dependent manner. However, LBP < 10 kDa had no effect on RAW264.7 cell viability (*p* > 0.05). This result may be due to the difference in monosaccharide composition and molecular weight between LBP > 10 kDa and LBP < 10 kDa. The above monosaccharide composition analysis has demonstrated that LBP > 10 kDa was rich in arabinose and galactose, those have been reported to activate macrophages through by TLR4 receptors on the cell surface [32]. In addition, polysaccharide ligands of TLR4 receptors with molecular weights ranging from 10 to 1000 kDa have been reported to have higher activity [13], which matched well with the average molecular weight of LBP > 10 kDa (27.71 kDa). LBP < 10 kDa (6.99 kDa) has low molecular weight, low levels of arabinose and galactose and low effect on RAW264.7 cells. This set of results indicates that the viability increasing effect of LBP on RAW264.7 cells is mainly contributed by the high molecular weight fraction (> 10 kDa). 

The above CCK-8 assay is an indirect method for evaluating the proliferative capacity of cells by detecting the dehydrogenase activity of all the viable cells. However, it does not ultimately prove whether the cells were proliferating. Therefore, we further performed the cell cycle assay and the CFSE distribution assays of RAW264.7 cells after treatment with different concentrations of LBP for 48 h. The cell cycle assay showed that LBP (200, 500 and 800 μg/mL) had no significant effect on cell phases (G1, S and G2/M) compared to untreated cells (Figure S7). The CFSE distribution assay is a powerful technique for the detection of cell proliferation by labeling of cells with 5-(and 6-) carboxyfluorescein diacetate succinimidyl ester (CFDASE) and then measuring the fluorescein fluorescence with flow cytometry. Cell proliferation usually results in a reduced fluorescence of cells and increased cell count. As shown in Figure 2c, the LBP treatment at different concentrations (200, 400 and 800 μg/mL) did not cause the reduce of fluorescein fluorescence in cells compared with the control group, suggesting that LBP did not induce the cell proliferation. Therefore, we further investigated the morphology of RAW264.7 cells after treatment with LBP by staining cells with dyes for cell membrane (FAM-WGA), mitochondria (Mito-Tracter) and nuclei (DAPI). The confocal imaging showed that after treatment with LBP > 10 kDa for 24 h, the size of cells, the average fluorescence intensity of mitochondria in cells and the average size of cell nucleus increased significantly compared with the control group (Figure 2d) (the quantitative comparison of mitochondrial fluorescence and nucleus size is shown in Figure S8). Many filopodia (as indicated by the arrows in Figure 2d) were observed around the cells after treatment with LBP > 10 kDa. However, LBP < 10 kDa did not show notable effect on the morphology of RAW264.7 cells, which is consistent with the experimental results of CCK-8 assay. It has been reported that macrophages are usually polarized after stimulation, resulting in their morphology change from a normal circle to a more specific dendritic structure [33,34,35]. This set of results suggests that the increase in the viability of RAW264.7 cells induced by crude LBP or LBP > 10 kDa cannot be attributed to the cell proliferation, and may due to the macrophage polarization. 

Macrophages are commonly polarized to two distinct subsets, M1 or M2 type. Classic M1 macrophages are pro-inflammatory and produce pro-inflammatory cytokines to promote pathogen responses and cytotoxic effects; whereas M2-type macrophages secrete anti-inflammatory cytokines and exert anti-inflammatory effects, including accelerated tissue repair and wound healing. CD86 is considered to be a marker of M1 macrophages, while CD206 is commonly used in the identification and screening of M2 macrophages [35,36]. In order to investigate the effect of LBP > 10 kDa on macrophage polarization, the changes of macrophage markers were detected by flow cytometry (Figure 2e,f and Figure S9). Compared with the control group, the expressions of CD86 and CD206 in LBP > 10 kDa group were significantly increased (*p* < 0.01). This result suggests that LBP > 10 kDa may maintain the balance of inflammation response by regulating macrophage differentiation to different subsets. 

**Figure 3:**
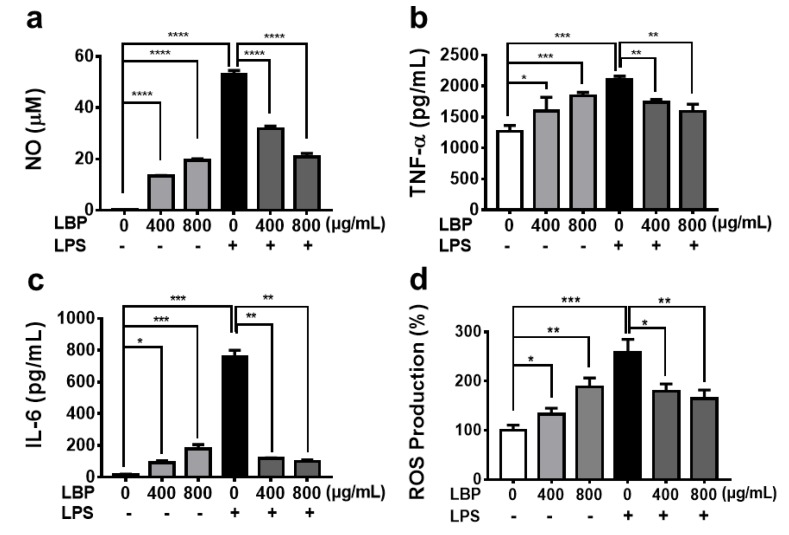
Inflammatory regulation of LBP on macrophages. The releases of NO (**a**), TNF-α (**b**) and IL-6 (**c**) from RAW264.7 cells after treatment by LBP > 10 kDa and LBP > 10 kDa + LPS (1 µg/mL). (**d**) The production of ROS in RAW264.7 cells after treatment by LBP > 10 kDa and LBP > 10 kDa + LPS (1 µg/mL). Results were expressed as means ± SD, *n* = 3. * *p* < 0.05, ** *p* < 0.01, *** *p* < 0.001, **** *p* < 0.0001.

The level of NO in macrophages was closely related to macrophage immune activity. NO was the main effector of activated macrophages to kill pathogenic microorganisms and tumor cells [35], but excessive NO also promoted the occurrence of inflammatory reactions and induced the secretion of inflammatory cytokines (e.g., TNF-α and IL-6). Lipopolysaccharide (LPS) is an activator of macrophages. It triggers macrophages to secret abundant cytokines, resulting in a strong inflammation response. Therefore, the levels of NO, TNF-α and IL-6 in RAW264.7 cell culture media were measured by ELISA after treatment with LBP and LBP + LPS. As shown in Figure 3a–c, LBP significantly promoted the release of NO, TNF-α and IL-6 from macrophages in a dose-dependent manner, but the extent of promotion effect was much lesser than that of LPS. In addition, LBP was also observed to have a significant inhibitory effect on LPS-induced NO, TNF-α and IL-6 overproduction. During phagocytosis, ROS in form of superoxide radical is produced as part of oxidative burst that helps in eradication of intracellular pathogen [34]. The ROS level in RAW264.7 cells was measured by flow cytometry assay. LBP was observed to significantly promote the production of ROS in a dose-dependent manner and also inhibit the LPS-induced ROS overproduction (Figure 3d and Figure S10). Other studies have shown that polysaccharides can enhance the body’s antibacterial, antiviral and antitumor capabilities, while avoiding the excessive production of inflammatory factors that damage tissues and organs [37,38]. This set of results suggests that appropriate amounts of LBP have the potential not only to be used as an immunostimulator to enhance the immune response, but also to prevent immune damage caused by excessive activation of macrophages. Of course, it needs to be further verified by animal experiments. 

### 2.4. The Uptake of Dye Labeled LBP by Macrophages

**Figure 4:**
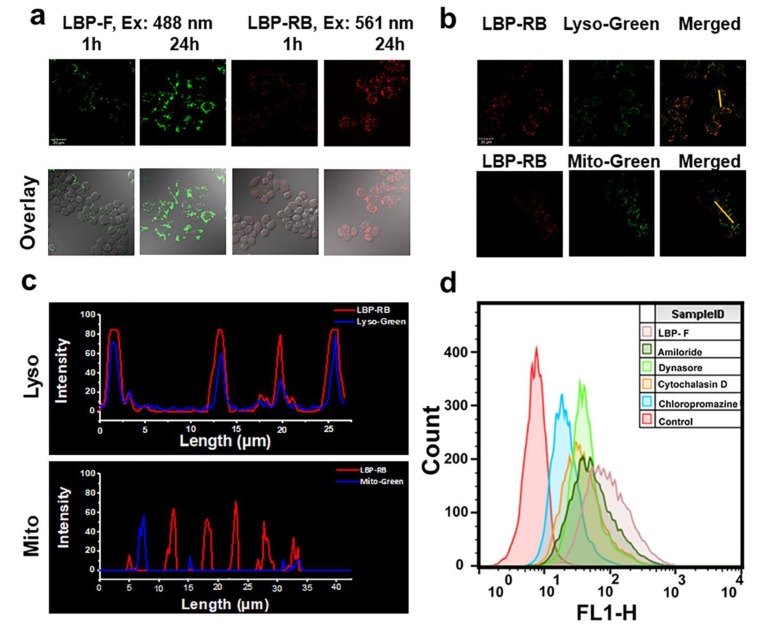
(**a**) Confocal imaging of RAW264.7 cells after incubation with 100 μg/mL LBP-F/LBP-RB after 1h and 24 h, (scale bar, 50 μm); (**b**) Confocal imaging of Raw264.7 cells stained with LBP-RB (100 μg/mL, λ_ex_ = 561 nm) and Lyso Tracker-Green (500 nM, λ_ex_ = 488 nm) or Mito Tracker-Green (100 nM, λ_ex_ = 488 nm), (scale bar, 50 μm); (**c**) fluorescence intensity profile of line regions in (**b**) of LBP-RB and Lyso-Green/Mito-Green; (**d**) the effects of inhibitors on the internalization of LBP-F measured by flow cytometry.

To understand the interaction of LBP with macrophages, we measured cellular uptake and intracellular localization of FITC and RBITC labeled LBP. The confocal imaging (Figure 4a) showed that both LBP-F and LBP-RB internalized into RAW264.7 cells in a time-dependent manner. The intracellular localization of LBP-RB was further investigated by co-staining with lysosome probe (Lyso Tracker Green) and Mitochondria probe (Mito Tracker Green) (Figure 4b,c). The confocal imaging revealed that LBP mainly localized in lysosomes. The interaction of LBP-F and LBP-RB was also tested on Caco-2, HeLa, LoVo, MCF-7 and MCF-7R cell lines, similar with RAW264.7 cells, LBP-F and LBP-RB could be internalized in all the tested cell lines and partially located in lysosomes (Figures S11–S15). The accumulation of LBP in lysosomes of all the tested cells suggests that the internalization of LBP may involve the endosomal pathway. Although LBP was able to enter all the cells tested, but it only had a noticeable effect on the RAW264.7 cells, suggesting the selective immunomodulatory effect of LBP. 

Due to the large molecular mass and high hydrophilicity of polysaccharides, it is difficult to enter cells by passive diffusion [39]. Some studies have shown that clathrin mediates the internalization process of polysaccharides [24]. The clathrin-dependent pathway includes three key steps: cell membrane infolding that depends on clathrin, vesicle formation that relies on dynamin, and endosome maturation. To investigate the endocytic pathway of LBP, four specific inhibitors for receptor-mediated cellular endocytosis pathway were used, including Chloropromazine (preventing the assembly and disassembly of clathrin lattices on cell membrane and endosomes) [40], Dynasore (inhibiting dynamin activity) [41], Cytochalasin D (disrupting actin filaments and inhibiting actin polymerization) [42], Amiloride (Epidermal sodium channel blocker) [43]. The flow cytometry assay (Figure 4d) showed that compared with the cellular uptake in the control group, all the inhibitors showed different degrees of inhibitory effect on the internalization of LBP, and the order of inhibition intensity was Chloropromazine > Cytochalasin D > Dynasore > Amiloride. The much stronger inhibitory effect of Chloropromazine than the others suggests that the clathrin-dependent endocytic pathway played relatively dominating roles in the endocytosis process of LBP. The confocal imaging further confirmed the effect of these inhibitors on cellular uptake of RBITC-labeled LBP (Figure S16). This set of results suggests that LBP was mainly internalized into macrophages through clathrin-dependent endocytic pathway, which is consistent with the accumulation of LBP in lysosomes. 

### 2.5. Transport of Dye Labeled LBP through Caco-2 Cell Monolayer

The human colon carcinoma cell line, Caco-2 could slowly differentiate to form a cell monolayer that possesses many functions of the small intestinal villus epithelium. Caco-2 cell monolayer has been widely used in the early screening of drug development and had become an important model to study the mechanism of drug absorption and transport [24]. The above results showed that LBP had no significant effect on the viability of Caco-2 cells, but it could be internalized into Caco-2 cells and partially locate in lysosomes (Figure S11), suggesting no toxicity of LBP to Caco-2 cells. It is possible that the internalized LBP might be transported out of Caco-2 cells. In order to investigate whether LBP can be absorbed by the small intestine, we further tested the transportation of LBP through Caco-2 cell monolayer. 

**Figure 5:**
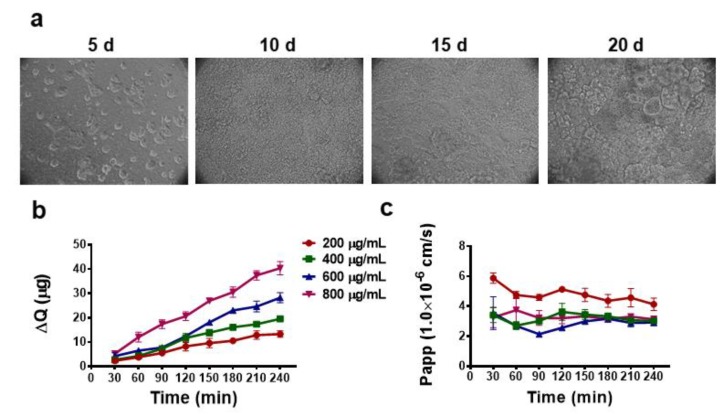
(**a**) The morphology of Caco-2 cells (Transwell plates) at different cultivated days (5 d, 10 d, 15 d, 21 d); (**b**) The accumulated transport amount curve of LBP-F through Caco-2 cell monolayer; (**c**) Papp values of LBP-F from AP to BL.

In order to obtain the cell monolayer for LBP absorption experiment, Caco-2 cells were grown on a polycarbonate film. The growth state of Caco-2 on the polycarbonate film was observed by IX71 inverted microscopy. As shown in the Figure 5a, after 20 days of culture, the cells formed a tight monolayer film. It has been reported that there is a good correlation between trans epithelial electrical resistance (TEER) values and single layer integrity [44,45]. In order to determine the integrity and compactness of the cell monolayers, the TEER values of the formed cell monolayers were monitored. The results showed that the TEER values increased with the growth time of cells. After 21 days of culture, the TEER values of all cell monolayers exceeded 400 Ω·cm^2^. Furthermore, a transport experiment with a hydrophilic paracellular marker (FITC) was performed to quantitatively determine the integrity of cell monolayer. There is a strong correlation between in vivo intestinal absorption and in vitro apparent permeability coefficient (Papp) for a variety of compounds. The well-absorbed drug (70~100%) usually has a Papp value higher than 10 × 10^−6^ cm/s. On the contrary, the poorly absorbed drug (0~20%) has a Papp value lower than 1 × 10^−6^ cm/s [43]. The total transmittance of FITC through the Caco-2 cell monolayer film was only 0.99 ± 0.58%, and the Papp value of FITC was 0.61 ± 0.36 × 10^−6^ cm/s, which was similar to the reference value reported in the literature [27]. This set of results suggests that the obtained Caco-2 cell monolayers met the Caco-2 model standard. Therefore, this model was used for subsequent LBP transport experiments.

To investigate whether LBP can be transported by epithelial cells, FITC labeled LBP was chased from the apical side to the basal side in a Caco-2 cell monolayer. As shown in Figure 5b, the transported LBP-F (200, 400, 600 and 800 μg/mL) was linear with time and did not reach saturation over a 240 min time frame. The Papp values of LBP-F through Caco-2 cell monolayer were measured to be in the range of 2.14 ± 0.17 × 10^−6^ cm/s~5.88 ± 0.35 × 10^−6^ (Figure 5c), suggesting that LBP-F was a well-absorbed biological macromolecule with an oral utilization rate of 20~70% [43]. In addition, the low concentration of LBP-F had a higher Papp value than the other groups, which may be due to a constant endocytosis rate and limited number of receptors on the cell surface that recognize polysaccharide macromolecules.

To investigate whether the uptake process is energy-dependent, the effect of LBP-F transport through Caco-2 cell monolayers at 4 °C and 37 °C was tested (Table 2). The Papp values of LBP-F (200 μg/mL) at 37 °C and 4 °C were measured to be 2.82 ± 0.44 × 10^−6^ cm/s and 0.46 ± 0.26 × 10^−6^ cm/s, respectively. The sharp decrease in the Papp value at 4 °C suggests that the absorption of LBP-F is an energy-consuming process. Since polysaccharides are macromolecular compounds, it is usually difficult to transport by active transport, and most macromolecules are absorbed and transported by clathrin-mediated endocytosis [24,46]. Therefore, the effect of the clathrin-mediated endocytosis inhibitor, chlorpromazine on the transport process was investigated. The Papp value of LBP-F (200 μg/mL) was measured to be 0.61 ± 0.18 × 10^−6^ cm/s after treating the Caco-2 cell monolayer with chlorpromazine (37 °C), suggesting that the absorption of LBP-F through small intestinal epithelium is also though the clathrin-mediated endocytosis and transport pathway.

The above results demonstrate at the cellular level the possibility of oral absorption of LBP through the small intestine and the possible absorption mechanism. However, the absorption process of drugs in animals is particularly complex, the further absorption experiments will be performed in animals to reveal the pharmacokinetic process of LBP.

## 3. Materials and Methods

### 3.1. Materials and Reagents

The dried mature fruits of *L. barbarum* were purchased from Ningxia Province, China. Lipopolysaccharides (LPS) was purchased from Sigma-Aldrich (St. Louis, MO, USA), Dimethyl sulphoxide (DMSO) was purchased from Panreac (Barcelona, Spain). Fluorescein isothiocyanate (FITC) and cytochalasin D were provided by J&K Chemicals (Beijing, China). Rhodamine B isothiocyanate (RBITC) was purchased from Heowns Biochem Technologies. Llc. (Tianjin, China). Chlorpromazine, Dynasore and Amiloride were purchased from Aladdin Bio-Chem Technology Co., Ltd. (Shanghai, China). Commercial ELISA kits for the analysis of tumor necrosis factor-α (TNF-α), interleukin-6 (IL-6) and NO detecting kit were bought from Beyotime Institute of Biotechnology (Jiangsu, China).

### 3.2. Preparation, Purification and Fractionation of LBP

The extraction method is illustrated in Figure 1. Briefly, the dried mature fruits of *L. barbarum* (250 g) were grounded into powder. In order to remove fat-soluble impurities, the powders were extracted twice with petroleum ether (boiling range, 60–90 °C). After centrifugation, the residue was extracted with 80% ethanol for two times to remove interfering components such as monosaccharides, oligosaccharides and glycosides. Then, the precipitate was extracted with deionized water and centrifuged at 8000 rpm for 5 min at 4 °C. The supernatant was collected and concentrated to an appropriate volume by a rotary vacuum evaporator and absolute ethanol was added into the supernatant to a final concentration of 80% (*v/v*) to precipitate for 12 h at 4 °C, and then lyophilized to obtain LBP. Considering the molecular structure and molecular weight of the polysaccharides may affect the biological activity [13], we used an ultrafiltration membrane with a molecular weight cut-off of 10 kDa to divide LBP into two components, which were designated as LBP < 10 kDa and LBP > 10kDa.

### 3.3. Structural Characterization of Polysaccharides

#### 3.3.1. Infrared Spectral Analysis of the Polysaccharides

The polysaccharide samples were mixed with KBr powder and pressed into pellets for FT-IR analysis, which was carried out using an Infrared Spectrometer TRENSOR 27 (Bruker Daltonics, Ettlingen, German) at the frequency range of 4000 to 400 cm^−1^.

#### 3.3.2. Determination of Molecular Weight

The molecular weight (Mw) of polysaccharide samples were determined by using a high-performance size elusion chromatography instrument equipped with a multi-angle laser light scattering and refractive index (HPSEC-MALLS-RID) system according to the method described by Sun et al. [47]. The HPSEC-MALLS-RID system consists of a pump (Waters515, Milford, USA), a MALLS detector (DAWN HELEOSII, Wyatt Technology, Santa Barbara, CA, USA), and a RI detector (Optilab T-rex, Wyatt Technology, Santa Barbara, CA, USA). LBP (10 mg) was dissolved in 1 mL of distilled water and filtered through a 0.22 μm filter. An amount of 20 μL of LBP sample solution was injected into the Shodex SB-806 HQ and Shodex SB-803 HQ chromatography columns (300 mm × 8.0 mm i.d. YMC Co. Ltd., Kyoto, Japan) and eluted with 0.02% NaN_3_ at a flow rate of 1.0 mL/min.

#### 3.3.3. Monosaccharide Composition Analysis

The glycosyl composition was analyzed using PMP pre-column derivation method as precious reported [23]. In briefly, the monosaccharide composition of LBP, LBP < 10 kDa and LBP > 10 kDa were determined after hydrolysis with 2 M TFA (trifluoroacetic acid) at 105 °C for 6 h. The analysis of PMP derivatives were performed on an Agilent 1100 series HPLC system consisted of a G1379A vacuum degasser, G1311A Quaternary pump, G1313A auto-sampler, and G1315B diode array detector. An amount of 20 μL of derivatives was injected onto a ZORBAX Eclipse XDB-C18 HPLC column (250 mm × 4.6 mm i.d. 5 μm) (Agilent, Santa Clara, CA, USA) operated at 30 °C, and eluted with a mixture of 0.1 M phosphate buffer (pH 6.7) and acetonitrile at a flow rate of 1.0 mL/min. UV detection wavelength was set at 250 nm.

#### 3.3.4. Analysis of Chemical Composition

Total sugar content was analyzed using the phenol-sulfuric acid method, using glucose as the reference. Protein content was determined by the Bradford’s method using bovine serum albumin as the standard.

#### 3.3.5. Elemental Analysis

Elemental analysis was carried out using a Flash EA1112 Elemental Analyser manufactured by Thermo Electron. The analyser was able to determine CHNS on dried samples.

### 3.4. Synthesis and Characterization of LBP-F/LBP-RB

In order to study the cellular uptake of LBP, Fluorescein and Rhodamine B were covalently linked to LBP by chemical method [48]. Briefly, 10 mg of LBP were dissolved in 2 mL of 0.5 M NaHCO_3_ buffer (pH 8.5), followed by 2.5 mg FITC were dissolved in 100 μL of DMSO and mixed well. The mixture was reacted in a shaker at room temperature for 24 h, The reactants were precipitated three times by 80% ethanol and purified by dialysis ((molecular cut-off size 500–1000 Da, Spectrum) for 3 days at 25 °C. Finally, the sample was freeze-dried to obtain LBP-F, which was further characterized by UV spectrophotometer UH5300 (HITACHI, Tokyo, Japan). In addition, LBP-RB was prepared by the same method with RBITC as the fluorescent labelling reagent.

To determine the labeling efficiency of LBP. FITC standard curve was obtained by plotting fluorescence intensity versus different concentrations of FITC standard solutions in water at ranging from 0.0001 to 1 μg/mL. Fluorescence intensity was measured using Hitachi FL-4600 fluorescence spectrophotometer (Kyoto, Japan) with excitation at 488 nm and emission at 520 nm. The fluorescence intensity of LBP-F was measured and the labeling degree was calculated with the regression equation, the whole process should avoid light. The fluorescence labeling degree of LBP-RB was measured by the same method but replace FITC with RBITC (Ex = 558 nm, Em = 585 nm).

### 3.5. Cell Culture and Cell Analysis

HeLa (cervical cancer), HepG2 (liver cancer), HEK293 (human embryonic kidney) and LoVo (colon cancer) cell lines were purchased from Typical Culture Preservation Commission Cell Bank, Chinese Academy of Sciences (Shanghai, China). MCF-7R (doxorubicin-resistant breast cancer) and A2780T (taxol-resistant ovarian cancer) cell lines were purchased from KeyGEN BioTECH Co. Ltd. (Nanjing, China). Caco-2 (human adenocarcinoma) and RAW264.7 (murine leukemic monocyte/macrophage) cell line was purchased from Cell Culture Center, Institute of Basic Medicine, Chinese Academy of Medical Sciences (Beijing, China). For cell culture, HeLa, HepG2 and LoVo cells were cultured in RPMI-1640 medium (1640, Gibco). RAW264.7 and HEK293 cells were cultured in Dulbecco’s Modified Eagle Medium (DMEM, Gibco). Caco-2 cell were cultured in Minimum Essential Medium (MEM, Gibco). All the basic media were supplemented with 10% fetal bovine serum (FBS, Gibco) and 1% penicillin/streptomycin (Corning). All the cells were routinely cultured at 37 °C, in a humidified atmosphere with 5% CO_2_.

#### 3.5.1. Cell Viability Assay

Cells in logarithmic growth phase were plated in 96-well plates at a density of 5 × 10^3^ cells per well in 100 μL of culture medium and were allowed to adhere for 24 h before treatment. Serial concentrations of each sample (200, 600, 1000, 2000 μg/mL) were then added (100 μL per well). After being treated for 48 h, the sample medium was removed, 110 μL of complete medium solution containing 10% CCK-8 was added to each well and incubated at 37 °C, 5% CO_2_ for 20 min. The absorbance at 450 nm was measured using a SpectraMax M5 microplate reader (Molecular Devices, Sunnyvale, CA, USA). The cell viability rates (VR) were calculated according to the Equation:

(1) $$\mathrm{VR}\;=\frac{A\;-{\;A}_{0}}{A_{S}-{\;A}_{0}}\times 100\%$$

where A is the absorbance of the experimental group, A_S_ is the absorbance of the control group and A_0_ is the absorbance of the blank group.

#### 3.5.2. Cell Proliferation Assay

Cell proliferation was investigated in two ways, CFSE distribution assay and Cell Cycle Analysis. CFSE distribution assay was carried out for monitoring cell proliferation. Shortly after, exponentially growing RAW264.7 cells (5 × 10^7^) were resuspended and washed twice with FBS-free medium, and then CFDA-SE stock solution was added into the suspension (final concentration: 10 μM) followed by incubation at 37 °C for 10 min in the dark, and then washed twice with five volumes of the culture medium. Cells were continually incubated in the culture medium at 37 °C for 10 min in the dark. After the supernatant was discarded, the CFSE-stained cells were grown in 12-well plates at the density of 1 × 10^5^ cells/well and allowed to attach to the bottom of the plates overnight. After being treated with different concentrations LBP (200, 500 and 800 μg/mL) for 48 h, the cell division was monitored by flow cytometry. For Cell Cycle Analysis, RAW264.7 cells (2 × 10^5^ cells/well) were cultured in 12-well plates for 12 h in a 37 °C humidified incubator with 5% CO_2_ and exposed to LBP at different concentrations (200, 500 and 800 μg/mL) for 48 h, then dispersed and washed with cold PBS three times, and fixed in 70% ethanol at 4 °C overnight. After being centrifuged at 2000 rpm for 5 min at 4 °C, the cells were resuspended in 0.5 mL of PBS containing 0.25% TritonX-100 and incubated on ice for 15 min. The cells were resuspended in 0.5 mL PBS containing 10 μg/mL RNase A and 20 μg/mL propidium iodide (PI) stock solution, transferred to FACS tubes and incubated at room temperature in the dark for 30 min. The cell cycle was analyzed by flow cytometry.

#### 3.5.3. Detection of Morphological Changes of Macrophages

RAW264.7 cells were plated in confocal dishes (35 mm × 12 mm, Φ 20 mm glass bottom) at a density of 2 × 10^5^ cells per dishes for 12 h in a 37 °C humidified incubator with 5% CO_2_. Then, the cells were incubated with 1 mL of fresh medium containing LBP < 10 kDa or LBP > 10 kDa (800 μg/mL) for 24 h. After removing the sample medium, cells were incubated with 2.5 µg/mL CytoFlamma Fluor WGA (a fluorescent probe for labeling cell membrane, excitation at 488 nm), 100 nM Mito-Tracker Green (a fluorescent dye for staining mitochondria, excitation at 488 nm) or 100 nM DAPI (a fluorescent dye for staining nucleus, excitation at 405 nm) at 37 °C for another 30 min respectively. Then, the stained cells were washed with cold PBS three times and observed by confocal imaging performed on an OLYMPUS FV3000-IX81 confocal microscope (Olympus Corporation, Tokyo, Japan). Confocal images were processed by Olympus FV10-ASW 4.2 viewer software (Olympus Corporation, Tokyo, Japan). The average fluorescence intensity of mitochondria in cells and the average size of cell nucleus were quantitatively analyzed by ImageJ software (V1.8.0).

#### 3.5.4. Induction Polarization of RAW264.7 Macrophages

Cells were treated as above (Section 3.5.2. Cell Cycle Analysis.). Macrophages were washed and dispersed in the PBS. Subsequently, cells were stained with fluorescein isothiocyanate (FITC)-conjugated anti-CD86 mAbs or phycoerythrin (PE)-labeled anti-CD206 mAbs (all from eBiosciences, San Diego, CA, USA), incubated at 4 °C for 30 min and then analyzed using flow cytometry (FACSCalibur, Becton Dickinson, Franklin Lakes, NJ, USA) and analyzed using FlowJo (V10).

#### 3.5.5. Measurement of Nitric Oxide (NO) and Cytokines Production

For measuring the effects of LBP on RAW264.7 cells: cells (2 × 10^5^ cells/well) were cultured in 12-well plates for 24 h, and incubated with different concentrations of LBP > 10 kDa for 48 h. For measuring the protective effect of LBP on LPS stimulation: RAW264.7 cells (2 × 10^5^ cells/well) were cultured in 12-well plates for 12 h, then incubated with different concentrations of LBP > 10 kDa for 12 h and finally incubated with LPS (1 μg/mL) for 48 h. The culture supernatant was collected, the NO in the culture supernatant were determined using the Griess reagent. The cytokines TNF-α, IL-6 released in the culture supernatant were measured by the ELISA Kits according to the instruction of manufacturers. The cells treated with only LPS (1 μg/mL) were used as positive control, and those cultured in DMEM medium were used as the negative control.

#### 3.5.6. Determination of ROS Production

Cells were treated as above (Section 3.5.5) and incubated with CellROX™ Deep Red Reagent (2.5 µM, Invitrogen) at 37 °C for 30 min, the intracellular ROS levels were measured with flow cytometry (BD Bioscience, Franklin Lakes, NJ, USA) and analyzed by FlowJo (V10). 

#### 3.5.7. Cellular Uptake and Localization of LBP

The internalization of LBP into Raw264.7, Caco-2, HeLa, LoVo, MCF-7 and MCF-7R was investigated by a Confocal laser spectrum microscope. Cells were plated in confocal dishes (35 mm × 12 mm, Φ 20 mm glass bottom) at a density of 2 × 10^5^ cells per dishes for 12 h in a 37 °C humidified incubator with 5% CO_2_. Cells were incubated with 1 mL of fresh medium containing LBP-F or LBP-RB (100 μg/mL) for 1h or 24 h. After incubation, the medium was removed, and cells were incubated with 500 nM Lyso Tracker Green (a fluorescent dye for staining Lysosomes, excitation at 488 nm) or 100 nM Mito Tracker Green (a fluorescent dye for staining mitochondria, excitation at 488 nm) at 37 °C for 30 min. Then the stained cells were washed with PBS three times and observed. Confocal images were obtained using a 100 × objective lens and the images were overlaid using Olympus FV10-ASW 4.2 viewer software (Olympus Corporation, Tokyo, Japan).

#### 3.5.8. Investigation of Endocytosis Pathways in RAW264.7 Cells

RAW264.7 cells (2 × 10^5^ cells/well) were cultured in 12-well plates for 24 h in a 37 °C humidified incubator with 5% CO_2_. Firstly, the Raw264.7 cells were pretreated with different transport inhibitors for 30 min: (I) 40 μM of Dynasore, (II) 40 μM of chlorpromazine, (III) 40 μM of Amiloride, (IV) 5 μM of Cytochalasin D. Then, the medium was removed, the cells were incubated with 100 μg/mL of LBP-FITC for 1 h. Subsequently, the RAW264.7 cells were washed three times with cold PBS and analyzed by FCM as described above. Because FITC is easily quenched under laser irradiation and strongly PH-dependent, and the background of cells is high at 488 nm, we further confirmed the effect of inhibitors on cellular uptake of LBP-RB with the help of laser confocal microscopy.

### 3.6. Transportation of LBP-F

#### 3.6.1. Establishment of Cellular Intestinal Model

An amount of 0.5 mL of Caco-2 suspension (2 × 10^5^ cells/mL) was seeded in the upper compartment (apical side, AP) of 12-well polycarbonate transwell plates (pore size: 0.4 μm, surface area: 1.12 cm^2^) and 1.5 mL MEM complete medium was added in the lower compartment (basal lateral, BL). Next, polycarbonate transwell plates were incubated at 37 °C in an atmosphere of 5% CO_2_ for 21 d in order to form confluent monolayer and the culture medium was changed every two days.

#### 3.6.2. Cell Monolayer Integrity Testing

The transepithelial electrical resistance (TEER) was measured with the Millicell ERS-2Volt ohmmeter (EMD Millipore, Billerica, MA, USA). The growth morphology of Caco-2 on Transwell polycarbonate membrane was recorded by IX71 inverted microscope (Olympus, Tokyo, Japan). Measurement of FITC transmission at 488 nm was measured by using a SpectraMax M5 microplate reader (Molecular Devices).

#### 3.6.3. Transport of the LBP-F through Caco-2 Cells Monolayer

Before the transport study, the culture medium in the 12-well polycarbonate transwell plates was discarded and the monolayer was washed three times with PBS, 0.5 mL of LBP-F solution with different concentrations was added to the apical side followed by addition of 1.5 mL of serum-free MEM medium solution to the basolateral side. At certain time intervals (every 30 min, 4 h totally), 200 μL solution was removed from basolateral side into 96-well plates. Fluorescence spectrometry quantification by FL-4600 fluorescence spectrometer (Hitachi Ltd., Tokyo, Japan) was set at λ_ex_ 488 nm, λ_em_ 520 nm. The content of LBP-F was calculated by FITC standard curve with a concentration range of 0–1 μg/mL.

The cumulative transmembrane transport (ΔQ) of LBP-F was calculated according to the equation:

ΔQ = C_n_V + ∑C_i_ΔV (i = 0, 1, 2, 3, …, n−1) (2)

where C_n_ is the drug concentration measured each time, V is volume of basolateral side (1.5 mL), and ΔV is the sample volume taken from the BL side each time (200 μL).

The apparent permeability coefficient (Papp) of LBP-F was calculated using the following equation:

Papp = (dQ/dt)/(A × C_0_) (3)

where dQ/dt is the cumulative transport rate (μg.min^−1^), A is the surface area of transwell plate (1.12 cm^2^) and C_0_ is the initial concentration of LBP-F.

#### 3.6.4. Effects of Temperature and Clathrin Inhibitor on the Absorption

To investigate whether the endocytosis pathways of LBP-F in the Caco-2 cells monolayer were energy-dependent, Caco-2 cell monolayers were incubated with LBP-F (200 μg/mL) at 4 °C for 2 h. For the endocytosis pathway inhibition experiment, the Caco-2 cells monolayers were pretreated with chlorpromazine endocytosis inhibitors at 37 °C for 30 min. Then, the Caco-2 cells monolayers were incubated with 200 μg/mL of LBP-F at 37 °C for another 2 h. The amount of LBP-F transported to the basolateral side was measured by a fluorescence spectrophotometer.

### 3.7. Data Handling and Presentation/Statistical Analysis

Data for quantification were acquired from individual experiments repeated at least three times, and expressed as the means ± SD. Statistical significance was calculated by GraphPad Prism 6 software (GraphPad Software, Inc., San Diego, CA, USA) with unpaired two-tailed t-tests and accepted by *p* < 0.05 (*), *p* < 0.01 (**), *p* < 0.001 (***), *p* < 0.0001 (****).

## 4. Conclusions

In this study, *Lycium barbarum* L. polysaccharide (LBP) was prepared by water extraction and ethanol precipitation, and was further separated into LBP > 10 kDa and LBP < 10 kDa fractions by ultrafiltration. The chemical properties of LBP were characterized (including molecular weight, monosaccharide composition, total sugar content, protein content and elemental analysis). LBP > 10 kDa fraction had the strong ability of enhancing the viability of RAW264.7 cells by inducing cell polarization, and had no notable effect on other tested tumor cell lines and normal cell lines. LBP > 10 kDa fraction was also found to have both pro-inflammatory and anti-inflammatory effects through regulating the secretion of NO, TNF-α, IL-6 and ROS in RAW264.7 cells. The cell uptake experiment showed that LBP could be internalized into RAW264.7 cells mainly through the clathrin-mediated endocytosis pathway and accumulated in lysosomes. LBP was also demonstrated to be transported through the Caco-2 cell monolayer via clathrin-mediated endocytosis. All these results suggest that LBP can be absorbed by the intestine and exert immunomodulatory effects in immune cells.

## Figures and Tables

**Table 1:** Structure Characterization of LBP fractions.

	LBP		LBP < 10 kDa	LBP > 10 kDa
	Peak 1	Peak 2		
**Molecular Characteristics**				
Mw	51.88	6.71	6.99	27.71
Mn	15.10	4.88	3.55	12.21
Mw/Mn	3.44	1.27	1.97	2.27
**Monosaccharide (Molar Ratio %)**				
Mannose	1.29		1.28	1.28
Glucose	30.61		47.27	11.68
Galacturonic acid	1.04		0.39	1.46
Galactose	7.03		1.20	13.37
Xylose	1.44		0.73	1.91
Arabinose	14.81		0.81	27.76
Fucose	0.38		1.81	0.62
Glucosamine	0.57		1.37	0.78
Rhamnose	0.91		0.82	1.67
Ribose	0.11		0.37	n.d.
**Chemical Compositions (%)**				
Protein	0.65 ± 0.14		0.47 ± 0.07	1.10 ± 0.03 *
Carbohydrates	26.58 ± 0.51		16.25 ± 0.24	29.69 ± 0.80 ***
**Elemental Analyses (%)**				
C	33.18 ± 0.05		30.08 ± 0.05	36.78 ± 0.01 ****
N	3.33 ± 0.02		3.38 ± 0.04	3.30 ± 0.06
H	4.78 ± 0.03		4.44 ± 0.02	5.41 ± 0.02 ****
S	0.73 ± 0.02		1.09 ± 0.04	0.96 ± 0.02

Peaks 1 and 2 were consistent with Figure S2. Mw: weight average molecular weight. Mn: number average molecular weight. n.d: not detected. Mean values ± standard deviation (*n* = 3). * Indicates significant difference between LBP > 10 kDa and LBP < 10 kDa (Student’s t-test, * *p* < 0.05, *** *p* < 0.001, **** *p* < 0.0001).

**Table 2:** Effect of temperature and clathrin inhibitor on the absorption of LBP-F.

Groups	ΔQ ug/cm^2^	Papp (×10^−6^ cm/s)
37 °C	8.11 ± 0.95	2.82 ± 0.44
4 °C	1.33 ± 0.55 ***	0.46 ± 0.26 **
Chlorpromazine hydrochloride (37 °C)	1.74 ± 0.39 ***	0.61 ± 0.18 **

Results were expressed as means ± SD, *n* = 3. ** *p* < 0.01, *** *p* < 0.001, vs. the 37 °C group.

## Supplementary Materials

The following are available online. Figure S1: FTIR analysis of LBP at a spectral range of 4000–400 cm^−1^; Figure S2: HPSEC chromatogram acquired by RI detector; Figure S3: UV–Vis spectroscopic analysis of LBP-F; Figure S4: UV–Vis spectroscopic analysis of LBP-RB; Figure S5: The standard curve of FITC (Ex = 488 nm, Em = 520 nm); Figure S6: The standard curve of RBITC (Ex = 558 nm, Em = 585nm); Figure S7: Cell cycle assay of RAW264.7 cells treated with different concentrations of LBP for 48 h; Figure S8: The average fluorescence intensity of mitochondria in cells (a) and the average size of cell nuclei (b); Figure S9: Percentage of CD86^+^/CD206^+^ RAW264.7 cells measured by flow cytometry after treatment by LBP >10 kDa; Figure S10: Flow cytometry assay of intracellular ROS levels after treatment by LBP > 10 kDa and LBP > 10 kDa + LPS (1 µg/mL); Figure S11: Confocal imaging of Caco-2 cells treated with LBP-F or LBP-RB and Lyso Tracker Green; Figure S12: Confocal imaging of LoVo cells treated with LBP-F or LBP-RB and Lyso Tracker Green; Figure S13: Confocal imaging of HeLa cells treated with LBP-F or LBP-RB and Lyso Tracker Green; Figure S14: Confocal imaging of MCF-7R cells treated with LBP-F or LBP-RB and Lyso Tracker Green; Figure S15: Confocal imaging of MCF-7 cells treated with LBP-F or LBP-RB and Lyso Tracker Green; Figure S16: Effects of various transport inhibitors on the internalization of LBP-RB in RAW264.7 cells.
