# Supplementary material for: Immunomodulatory Effects of Lycium barbarum Polysaccharide Extract and Its Uptake Behaviors at the Cellular Level
Source: Molecules. 2020 Mar 16;25(6):1351. doi: 10.3390/molecules25061351 (PMC7145302; doi:10.3390/molecules25061351)
Supplement: Supplementary file 1 [file molecules-25-01351-s001.pdf]

Immunomodulatory Effects of *Lycium Barbarum* Polysaccharide Extract and Its Uptake Behaviors at the Cellular Level

Le Feng [1,2], Xiao Xiao [2,3], Jing Liu [2,3], Junyan Wang [2,3], Nan Zhang [2,3], Tao Bing [2,3], Xiangjun Liu [2,3], Ziping Zhang [1,*] and Dihua Shangguan [2,3,*]

[1] Key Lab of Ministry of Education for Protection and Utilization of Special Biological Resources in Western China, School of Life Sciences, Ningxia University, Yinchuan, 750021, China
[2] Beijing National Laboratory for Molecular Sciences, Key Laboratory of Analytical Chemistry for Living Biosystems, CAS Research/Education Center for Excellence in Molecular Sciences, Institute of Chemistry, Chinese Academy of Sciences, Beijing, 100190, China
[3] University of Chinese Academy of Sciences, Beijing, 100049, China
* To whom correspondence should be addressed: e-mail: zipingzhang@163.com, sgdh@iccas.ac.cn

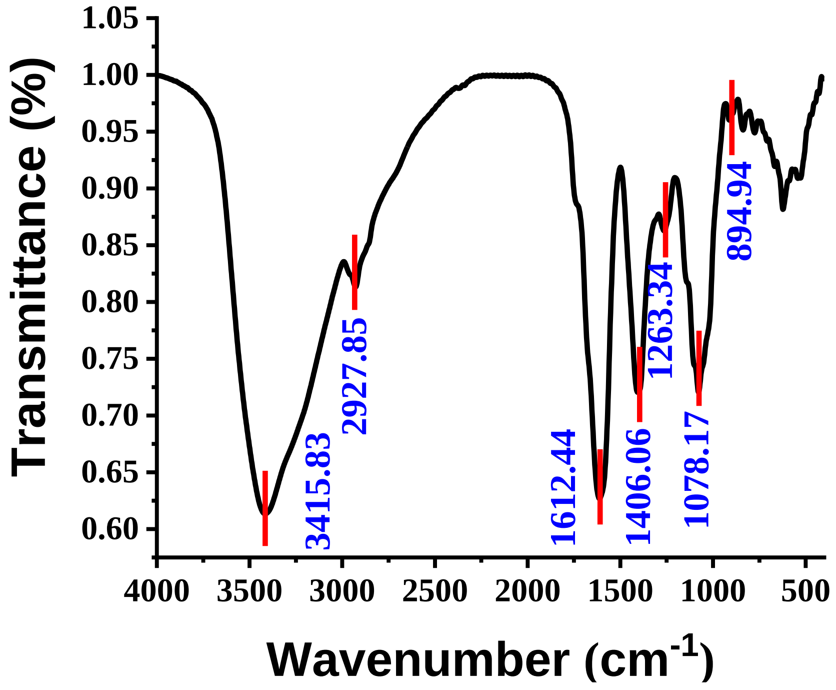

Figure S1. FTIR analysis of LBP at a spectral range of 4000–400 cm^{-1}.

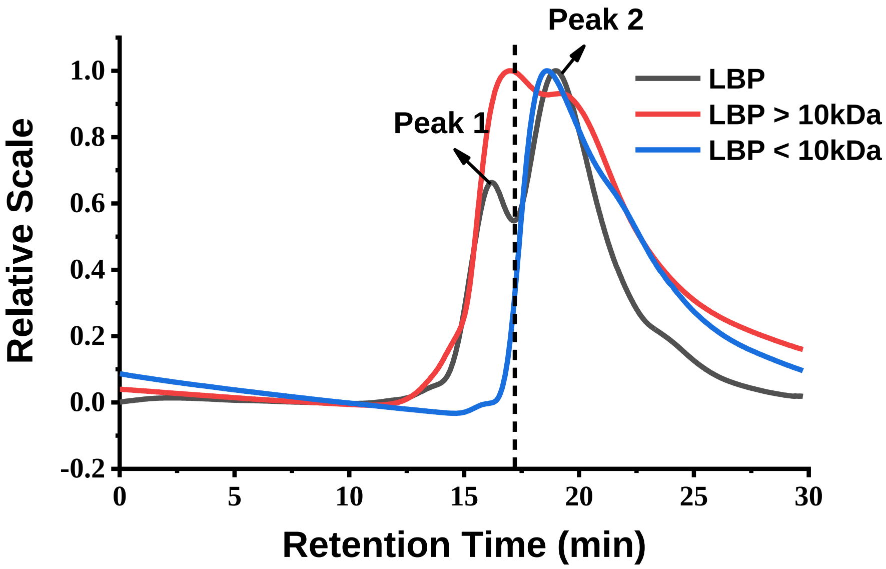

Figure S2. HPSEC chromatogram acquired by RI detector.

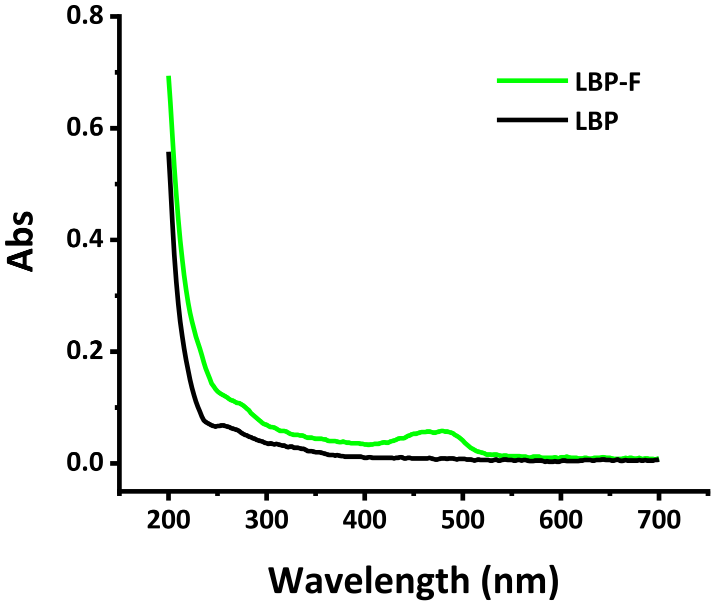

Figure S3. UV–Vis spectroscopic analysis of LBP-F.

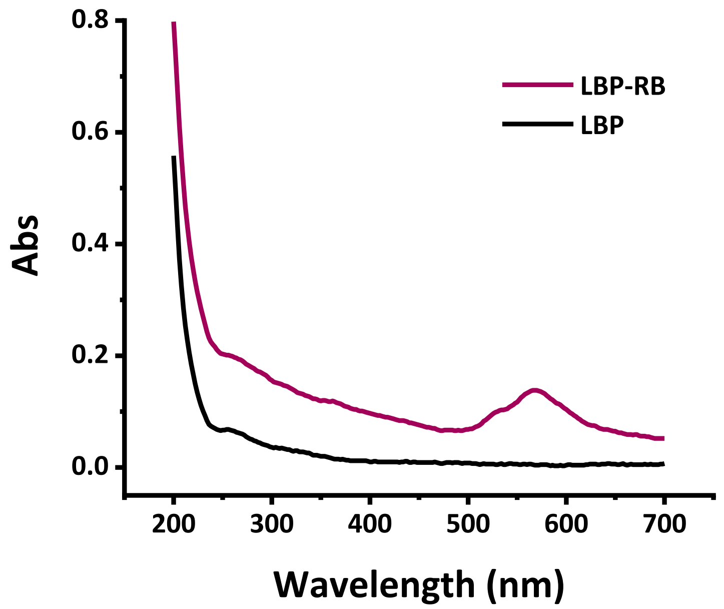

Figure S4. UV–Vis spectroscopic analysis of LBP-RB.

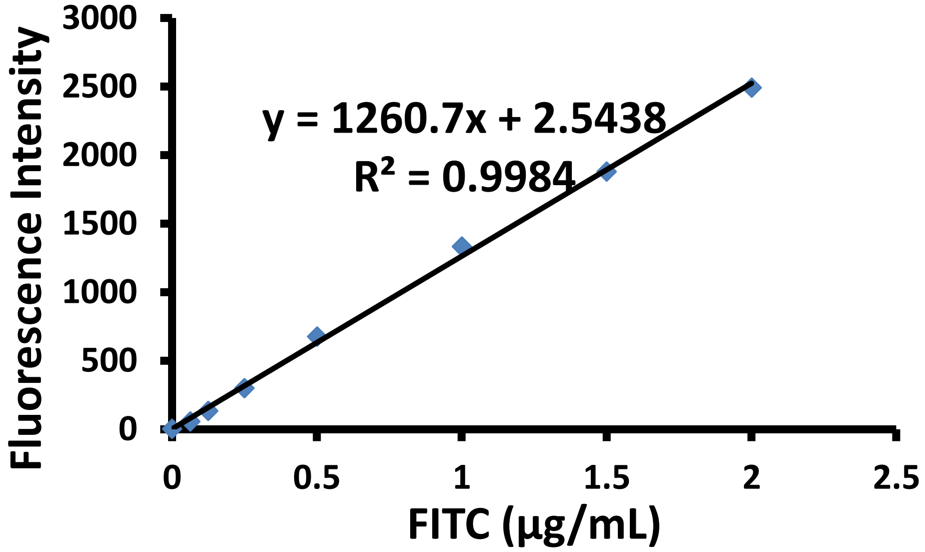

Figure S5. The standard curve of FITC (Ex=488 nm, Em=520 nm).

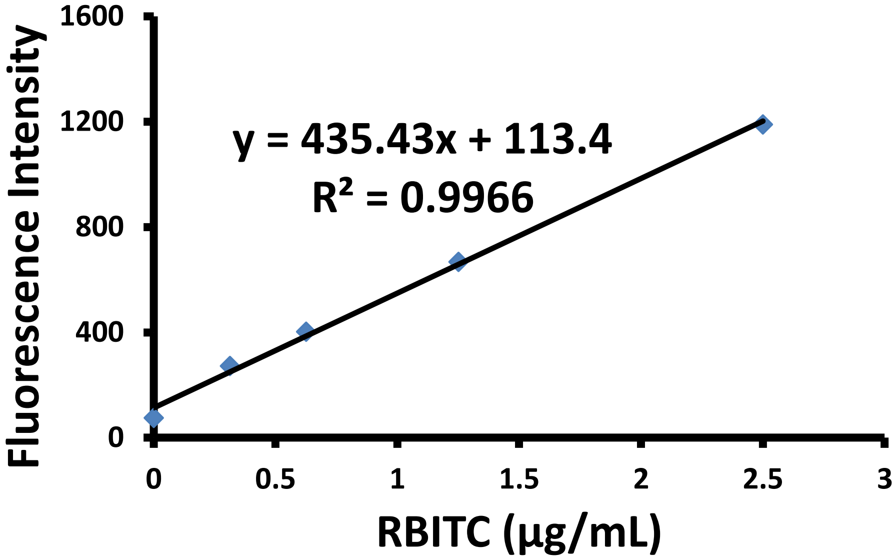

Figure S6. The standard curve of RBITC (Ex=558 nm, Em=585nm).

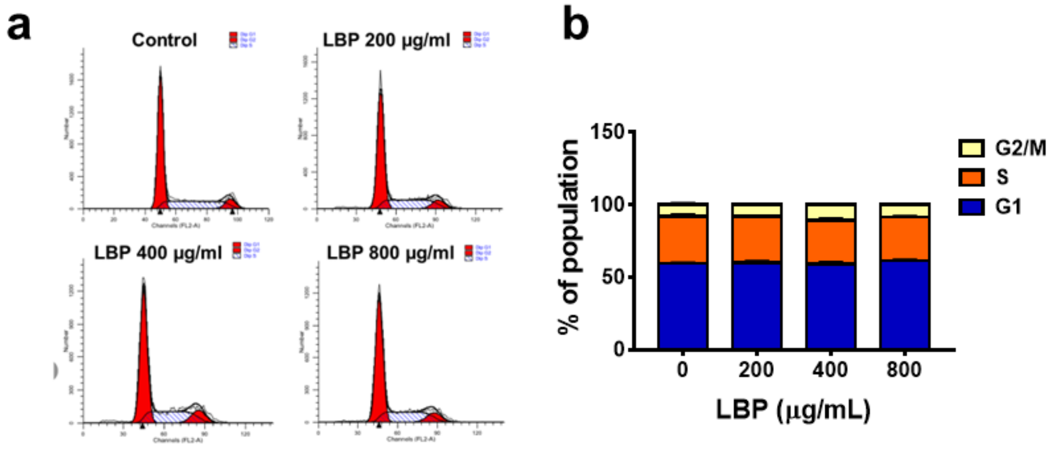

Figure S7. Cell cycle assay of RAW264.7 cells treated with different concentrations of LBP for 48 h. (a) The representative histograms; (b) The quantitative analysis of cell population in different phases.

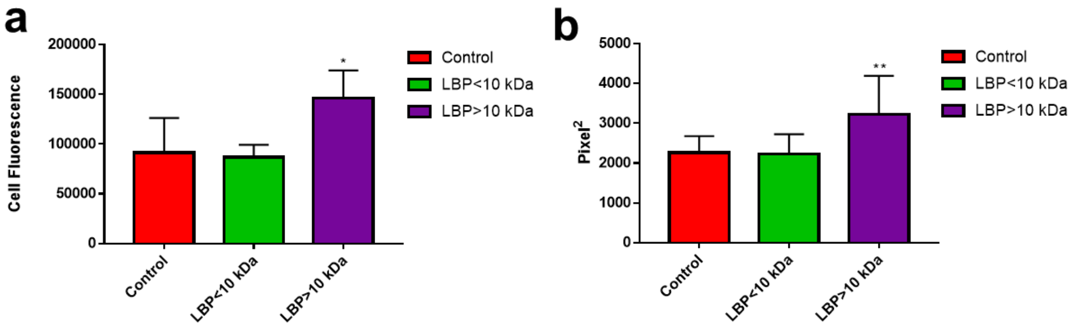

Figure S8. The average fluorescence intensity of mitochondria in cells (a) and the average size of cell nuclei (b).

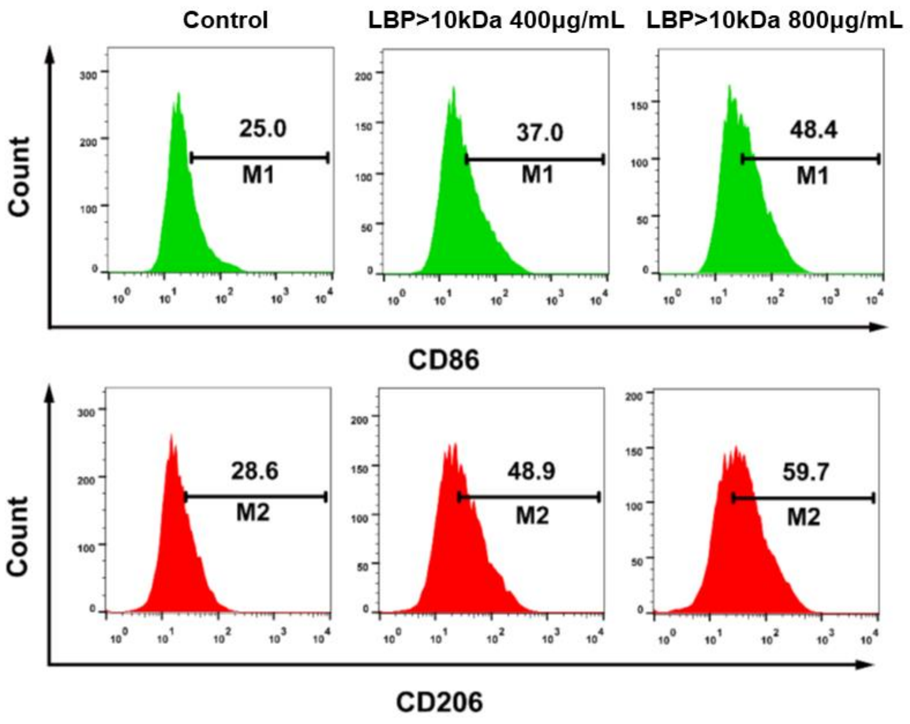

Figure S9. Percentage of CD86+ /CD206+ RAW264.7 cells measured by flow cytometry after treatment by LBP >10 kDa.

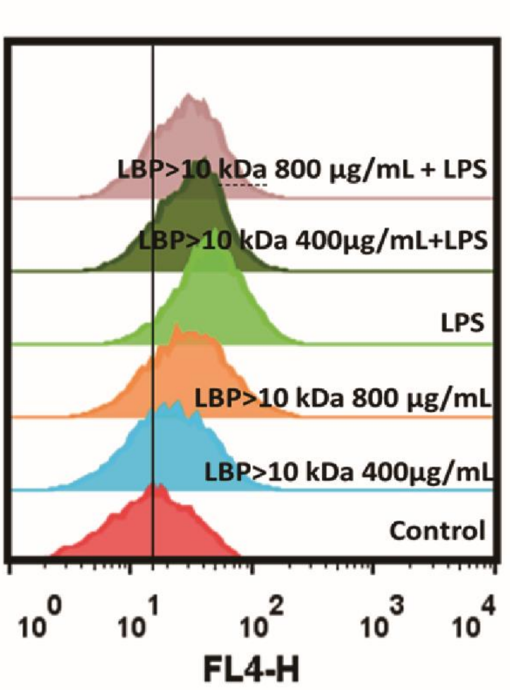

Figure S10. Flow cytometry assay of intracellular ROS levels after treatment by LBP > 10 kDa and LBP > 10 kDa +LPS (1 µg/mL).

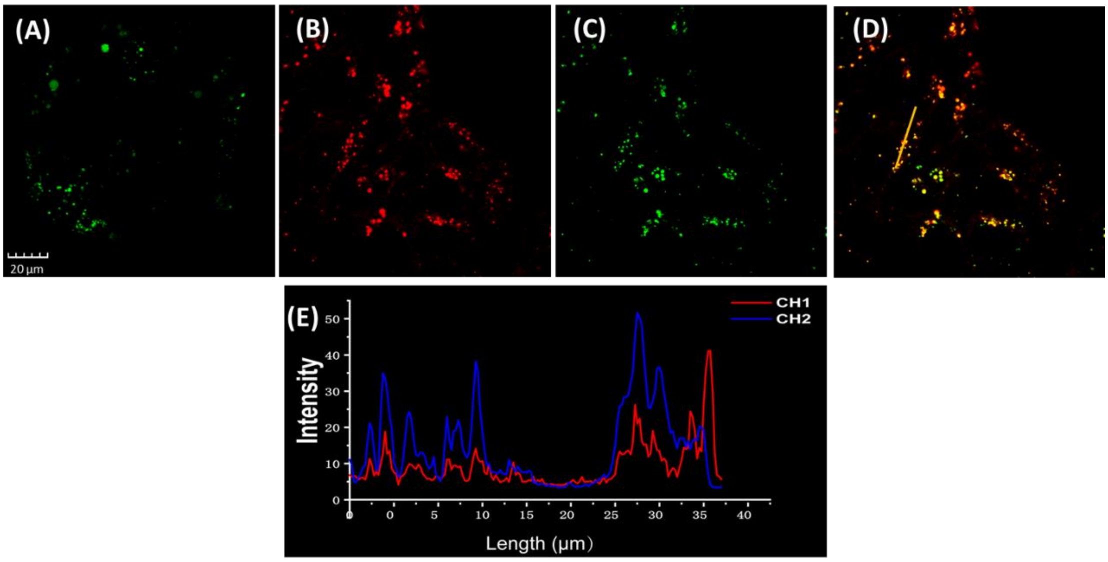

Figure S11. Confocal imaging of Caco-2 cells treated with LBP-F or LBP-RB and Lyso Tracker Green. The cells were incubated with LBP-F (100 μg/mL) or LBP-RB (100 μg/ml), and Lyso Tracker Green (1 μM) for 30 min at 37 °C. (A) Confocal image from LBP-F on channel 1(λex = 488 nm). (B) Confocal image from LBP-RB on channel 2 (λex =561 nm). (C) Confocal image from Lyso Tracker Green on channel 1 (λex = 488 nm). (D) Merged image of B and C. (E) fluorescence intensity profile of line regions in (D) of LBP-RB and Lyso Tracker Green, Scale bars: 20 μm.

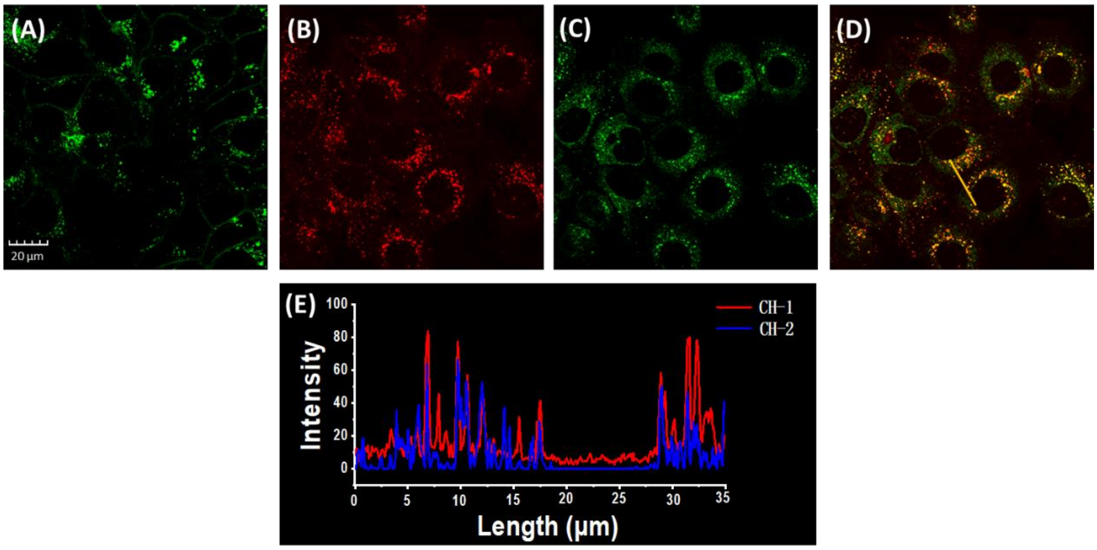

Figure S12. Confocal imaging of LoVo cells treated with LBP-F or LBP-RB and Lyso Tracker Green. The cells were incubated with LBP-F (100 μg/mL) or LBP-RB (100 μg/ml), and Lyso Tracker Green (1 μM) for 30 min at 37 °C. (A) Confocal image from LBP-F on channel 1(λex = 488 nm). (B) Confocal image from LBP-RB on channel 2 (λex =561 nm). (C) Confocal image from Lyso Tracker Green on channel 1 (λex = 488 nm). (D) Merged image of B and C. (E) fluorescence intensity profile of line regions in (D) of LBP-RB and Lyso Tracker Green, Scale bars: 20 μm.

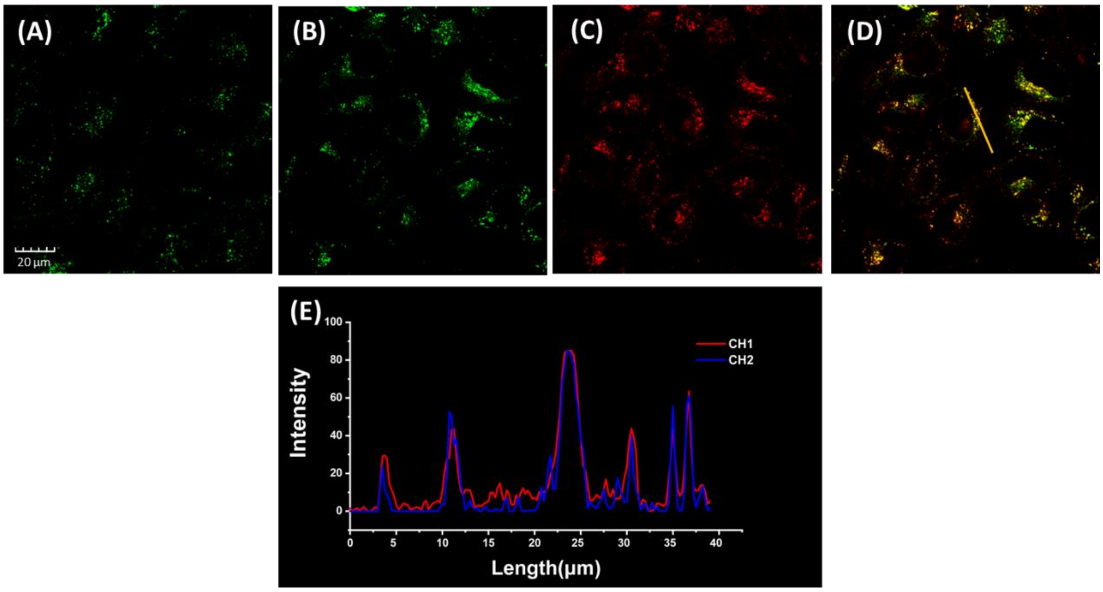

Figure S13. Confocal imaging of HeLa cells treated with LBP-F or LBP-RB and Lyso Tracker Green. The cells were incubated with LBP-F (100 µg/mL) or LBP-RB (100 µg/ml), and Lyso Tracker Green (1 µM) for 30 min at 37 °C. (A) Confocal image from LBP-F on channel 1(λex = 488 nm). (B) Confocal image from LBP-RB on channel 2 (λex =561 nm). (C) Confocal image from Lyso Tracker Green on channel 1 (λex = 488 nm). (D) Merged image of B and C. (E) fluorescence intensity profile of line regions in (D) of LBP-RB and Lyso Tracker Green, Scale bars: 20 µm.

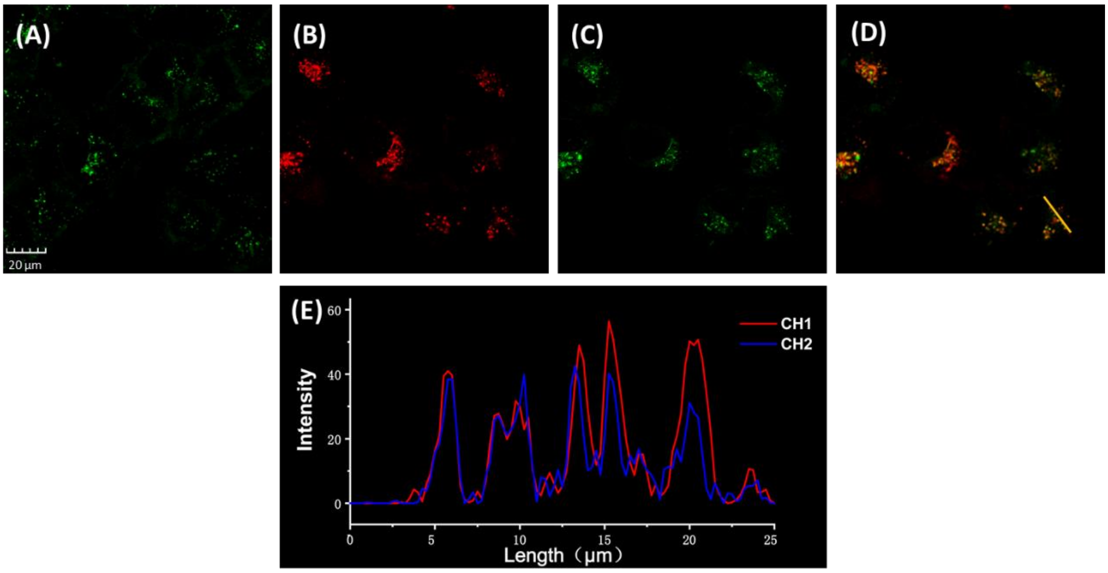

Figure S14. Confocal imaging of MCF-7R cells treated with LBP-F or LBP-RB and Lyso Tracker Green. The cells were incubated with LBP-F (100 µg/mL) or LBP-RB (100 µg/ml), and Lyso Tracker Green (1 µM) for 30 min at 37 °C. (A) Confocal image from LBP-F on channel 1(λex = 488 nm). (B) Confocal image from LBP-RB on channel 2 (λex =561 nm). (C) Confocal image from Lyso Tracker Green on channel 1 (λex = 488 nm). (D) Merged image of B and C. (E) fluorescence intensity profile of line regions in (D) of LBP-RB and Lyso Tracker Green, Scale bars: 20 µm.

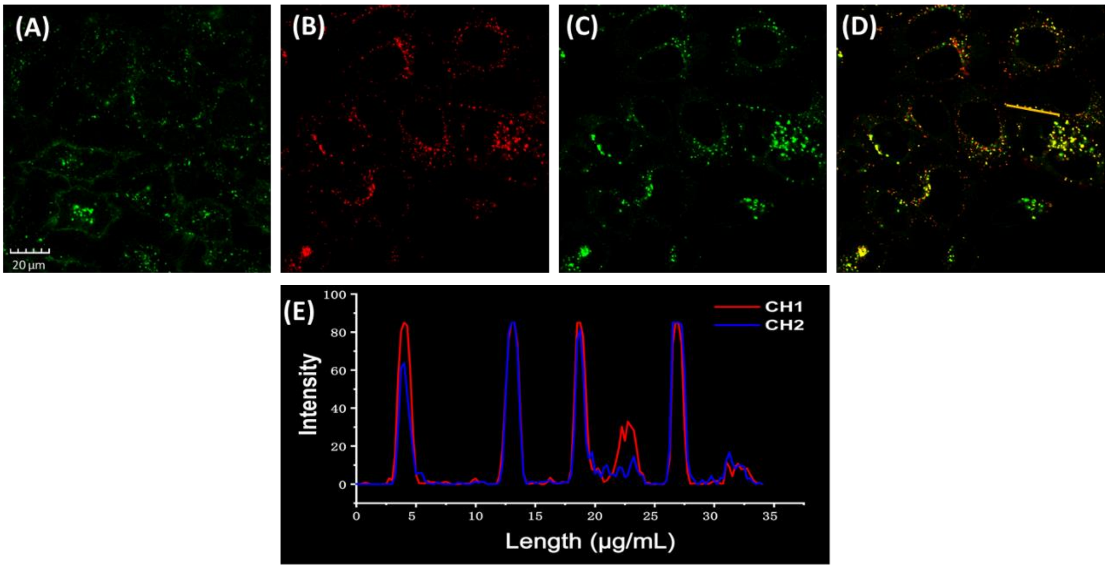

Figure S15. Confocal imaging of MCF-7 cells treated with LBP-F or LBP-RB and Lyso Tracker Green. The cells were incubated with LBP-F (100 μg/mL) or LBP-RB (100 μg/ml), and Lyso Tracker Green (1 μM) for 30 min at 37 °C. (A) Confocal image from LBP-F on channel 1(λex = 488 nm). (B) Confocal image from LBP-RB on channel 2 (λex =561 nm). (C) Confocal image from Lyso Tracker Green on channel 1 (λex = 488 nm). (D) Merged image of B and C. (E) fluorescence intensity profile of line regions in (D) of LBP-RB and Lyso Tracker Green, Scale bars: 20 μm.

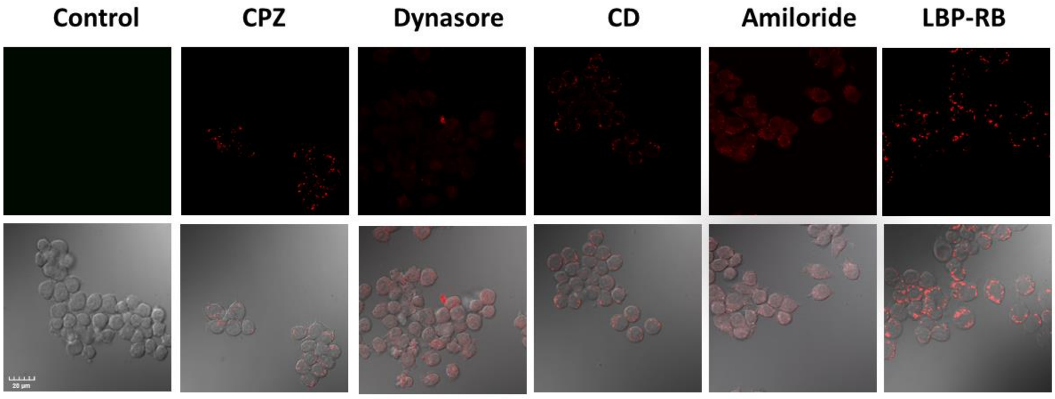

Figure S16. Effects of various transport inhibitors on the internalization of LBP-RB in RAW264.7 cells (Ex=561 nm). Scale bars: 20 μm.
